# Indolent nodal T follicular helper cell lymphomas—A case series

**DOI:** 10.1038/s41408-024-01163-y

**Published:** 2024-11-26

**Authors:** Jie Wang, Chun En Yau, Chen Ee Low, Mohamed Haniffa Bin Hasan Mohamed, Chee Leong Cheng, Jadee L. Neff, Jing Quan Lim, Soon Thye Lim, Jason Yongsheng Chan, Choon Kiat Ong, Valerie Shiwen Yang

**Affiliations:** 1 Hematologic Malignancies and Cellular Therapy, 2400 Pratt St, Ste 5000, Durham, NC 27705 USA; 2 Yong Loo Lin School of Medicine, National University of Singapore, 10 Medical Dr, Singapore, 117597 Republic of Singapore; 3 Division of Medical Oncology, National Cancer Centre Singapore, 30 Hospital Boulevard, Singapore, 168583 Republic of Singapore; 4 Department of Anatomical Pathology, Singapore General Hospital, Outram Rd, Singapore, 169608 Republic of Singapore; 5 Department of Pathology, Duke University Medical Center, 10 Duke Medicine Cir, Durham, NC 27710-1000 USA; 6 Division of Cellular and Molecular Research, National Cancer Centre Singapore, 30 Hospital Boulevard, Singapore, 168583 Republic of Singapore; 7 Oncology Academic Clinical Program, Duke-NUS Medical School, 8 College Road Singapore, Singapore, 169857 Republic of Singapore; 8 Director’s Office, National Cancer Centre Singapore, 30 Hospital Boulevard, Singapore, 168583 Republic of Singapore; 9 Office of Education, Duke-NUS Medical School, 8 College Road Singapore, Singapore, 169857 Republic of Singapore; 10 Cancer and Stem Cell Biology Program, Duke-NUS Medical School, 8 College Road Singapore, Singapore, 169857 Republic of Singapore; 11 Translational Precision Oncology Laboratory, Institute of Molecular and Cell Biology (IMCB), Agency for Science, Technology and Research (A*STAR), 61 Biopolis Drive, Proteos, Singapore, 138673 Republic of Singapore

**Keywords:** Lymphoma, Translational research

Letter to the Editor,

Angioimmunoblastic T-cell lymphoma (AITL) originates from T follicular helper (TFH) cells and is widely considered to be an aggressive T-cell neoplasm. Cure rates are low, and relapses are common, with a median overall survival of only three years [1–3]. In the 2022 World Health Organization classification [4], the term nodal TFH (nTFH) cell lymphomas unites the subtypes of angioimmunoblastic type, follicular type and a not otherwise specified (NOS) category [5]. These entities, distinguished by histopathological features, have considerable overlap and potential for inter-observer variability. Despite the apparent biological heterogeneity, all entities are treated aggressively according to current treatment guidelines [1, 6]. nTFH cell lymphomas with an indolent nature exist [7], but such an entity has never formally been described.

We present a case series of patients from the National Cancer Centre Singapore and Duke Cancer Institute with nTFH cell lymphomas who survived over a year despite declining systemic chemotherapy. Their diagnoses were confirmed after an independent review by expert hematopathologists at both institutions. Key characteristics are summarized in Table 1. The histological samples were classified into three patterns - Pattern 1: follicular hyperplasia, pattern 2: regressed follicles, pattern 3: effaced architecture, lacking follicles [8–10]. Examples of pathological investigations are presented in Fig. 1.

**Table 1:** Clinical and pathological characteristics of included patients.

Case number	Sex/Age at diagnosis	Ann Arbor Stage	Pattern	T-cell phenotype or TFH phenotype	Epstein-Barr virus-encoded RNA (EBER)	Management	Follow-up	Status at last follow-up
Case 1	Male/ 64	I	AITL patterns 1 & 2	CD4 + , PD1 + , ICOS+	Negative	Surgical excision of single site of involvement and observation	5 years	Alive
Case 2	Male/ 69	II	AITL pattern 1	CD4 + , PD1 + , ICOS+	Negative	Observation	5 years	Alive
Case 3	Female/ 70	I	AITL pattern 1	CD4 + , CD10 + , PD1 + , ICOS+	Scattered positive	Excisional LN biopsy of single site of disease	5 years	Alive
Case 4	Female / 72	I	AITL pattern 1	PD1 + , ICOS+	EBER+ in large B cell immunoblasts	Observation	3 years	Alive
Case 5	Female / 67	III	AITL pattern 1	CD4+ PD1 + ICOS +	Positive	Observation	6 years	Alive
Case 6	Female / 42	III	AITL pattern 1	CD4+ PD1 + ICOS +	Positive	Observation	5 years	Alive
Case 7	Female / 52	II	AITL patterns 1 & 2	ICOS+ PD1+	Positive	Prednisolone and paracetamol	5 years	Alive
Case 8	Female / 74	IV	AITL pattern 1	CD4 + CD5-^a^	Positive	Prednisolone for concomitant diagnosis of ILD	6 years	Deceased
Case 9	Female / 78	II	AITL pattern 3	CD4 CD10+ BCL6+ PD1+	Positive	Prednisolone	22 months	Deceased

^a^CD4 + T-cells with CD5 loss are present in peripheral areas of germinal centers in keeping with a neoplastic Tfh population. Prominent high endothelial venules and presence of focal areas of follicular dendritic cell expansion are seen.

**Fig. 1:**
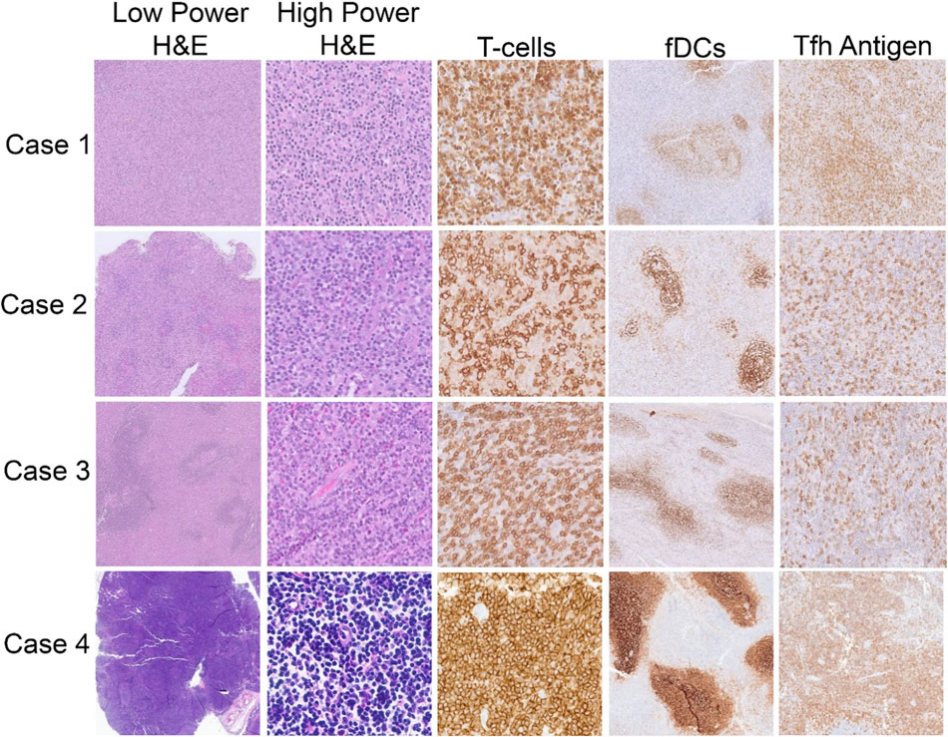
Examples of pathological visualization of available AITL cases. Low-power images (left column) of biopsied lymph nodes show varying degrees of architectural effacement by a paracortical infiltrate. High-power images (2^nd^ to left column) of the paracortical infiltrate show atypical mature lymphocytes. T-cell-specific immunohistochemical stains (Case 1, BetaF1; Case 2, CD2; Cases 3 and 4, CD3) highlight the T-cell atypia. Follicular dendritic cell meshworks (fDCs) are focally mildly expanded in all cases (CD21 immunostain). T follicular helper cell (Tfh) antigens were expressed in all cases, illustrated by ICOS (Cases 1-3) and PD1 (Case 4) immunostains.

Case 1 describes a 64-year-old man who presented with left-sided facial swelling in 2018. He underwent left parotidectomy in 2020, with histological patterns 1 and 2. Positron emission tomography-computed tomography (PET-CT) showed no other sites of disease. Bone marrow assessment was negative for involvement. He has now been well without treatment for five years.

Case 2 is a 69-year-old man, who was incidentally found to have enlarged cervical lymph nodes on MRI. In December 2017, an excisional biopsy of a right cervical lymph node showed AITL pattern 1. Bone marrow involvement was negative. Cross-sectional imaging every six months performed for the following two years was stable with decreased lymphadenopathy in June 2020. He has been without treatment for over five years.

Case 3 is a 70-year-old woman who presented with a painless submental mass and diagnosed with AITL by excisional biopsy in March 2018. PET-CT showed no other sites of disease. Bone marrow involvement was negative. She was observed and PET-CT in April 2019 was negative for recurrence. She has been without treatment for five years.

Case 4 is a 73-year-old woman with a longstanding history of thyroiditis and goiter. In April 2021, she underwent thyroidectomy due to a progressively enlarging goiter with compressive symptoms. Pathology showed thyroid gland and cervical lymph node involvement by PTCL TFH. Epstein-Barr virus-encoded RNA (EBER) was scattered positive. PET-CT scan did not show other sites of involvement by disease. Bone marrow biopsy was not performed. Follow-up PET-CT scan in January 2022 was negative for metabolically active disease. She has been well for three years without treatment.

The first four patients had excised localised lymphadenopathy that did not have recurrence. However, aggressive lymphomas are presumed systemic and still treated with multi-agent chemotherapy in general.

Case 5 is a 67-year-old asymptomatic woman who presented in 2017 with axillary lymphadenopathy incidentally found on routine mammogram. Excisional axillary lymph node biopsy in March 2017 showed AITL pattern 1. EBER immunohistochemistry (IHC) was positive. CT showed borderline enlarged lymph nodes in the neck, axilla, pulmonary hilum, abdomen, pelvis and inguinal regions. The spleen was also borderline enlarged. Bone marrow biopsy showed mild atypical lymphocytosis without definite involvement by lymphoma. Surveillance scans for two years from diagnosis showed waxing and waning lymph nodes. Most recent CT in March 2020 showed resolution of all lymphadenopathies. She has now been in follow-up for six years.

Case 6 is a 41-year-old woman who presented in August 2018 for five months of enlarged left axillary lymph node. Excisional biopsy showed AITL pattern 1. At the same time, there were overwhelming areas of necrotizing granulomatous inflammation, concerning for concomitant tuberculoid lymphadenitis. EBER IHC was negative. PET-CT showed multiple fludeoxyglucose-avid lymph nodes in the neck, and multiple enlarged lymph nodes above and below the diaphragm. She was treated for tuberculosis for six months. PET-CT in February 2020 showed decrease in lymphadenopathy. She has been without treatment for five years. The first six patients did not experience any B symptoms.

Case 7 is a 53-year-old woman who presented in 2018 with slowly enlarging right neck lymph node over five years. She had night sweats and a weight loss of 12 kilograms over five months, treated symptomatically with prednisolone and paracetamol. Excisional biopsy of the cervical lymph node in September 2018 showed AITL patterns 1 and 2. CT showed cervical lymphadenopathy, without significantly enlarged lymph nodes elsewhere. Bone marrow biopsy was not performed. EBER IHC was positive. Follow-up CT scans in May 2019 and January 2020 showed stable disease. CT in August 2020 shows a resolution of adenopathy. She is well and last follow-up was in May 2023, over five years after diagnosis.

Case 8 is a 74-year-old woman with interstitial lung disease (ILD). In 2011 she presented with progressive dyspnea and CT chest showed enlarged mediastinal lymph nodes. PET-CT scan showed lymphadenopathy above and below the diaphragm. A supraclavicular lymph node biopsy showed AITL pattern 1. Bone marrow biopsy showed focal lymphoid aggregates of B and T-cells with features suggestive of a lymphoproliferative disorder. EBER IHC was positive. She was treated for ILD with prednisolone from September 2011 to March 2015. Restaging PET-CT at that time showed no evidence of lymphadenopathy. She did not pursue any further oncologic treatment, given her comorbidities. She died in February 2017, 6 years from diagnosis, from complications unrelated to AITL.

Case 9 is a 78-year-old woman who presented in 2016 for enlarged cervical lymphadenopathy for one year. CT showed bilateral cervical lymphadenopathy and left axillary lymphadenopathy. The patient declined bone marrow staging. Excisional biopsy of a left cervical lymph node showed AITL pattern 3. She was offered palliative treatment with chlorambucil and prednisolone, but she opted to receive only prednisolone which she started in August 2016. CT in January 2017 showed a partial response and by May 2017, she was weaned off prednisolone. However, in July 2017, she was restarted on prednisolone when cervical lymphadenopathy was noted on physical exam. In June 2018, restaging CT showed progressive disease with new lymphadenopathy and splenomegaly. She died in July 2018, 22 months after diagnosis.

Even though steroids alone are generally insufficient to control aggressive lymphomas long-term, follow-up of cases 7 and 8 showed no progression of disease, while case 9 exhibited a partial response. Only cases 4 and 8 had comorbidities. No patients used alternative medicine therapies.

To our knowledge, this is the first case series describing clinical outcomes of never-treated nTFH cell lymphoma. Current clinical treatment guidelines recommend multiagent chemotherapy in the first-line regardless of stage of disease, and clinical presentation. In our case series, many of the patients had a limited burden of disease, and all declined chemotherapy. Target enrichment sequencing was performed in three subjects where extant biopsy material was available and none had mutations in TET2, DNMT3A, or IDH2, three commonly mutated genes in nTFH cell lymphomas. TET2 and DNMT3A mutations occur in about 80% and 30% of AITL cases, respectively, disrupting DNA methylation [11]. These mutations have been shown to influence patients’ survival [11]. Mutations in the IDH2 gene are found in 20% of cases, influencing oncometabolite production and histone methylation [12]. Targeted genes screened for mutations and mutations identified in formalin-fixed paraffin-embedded tumour tissue from these subjects are in Supplementary Tables 1 and 2. In our study, coverages of 117.2x, 105.4x and 253.7x were achieved for cases 1–3, respectively. Nevertheless, the depth of sequencing was variable. Data on estimated tumour purity is included in Supplementary Table 3. We used a variant-depth cutoff of ≥3, and a variant allele frequency of ≥5%. A mean sequencing depth of >100x would be sufficiently sensitive to represent variants of such attributes. Due to the limited samples from only three patients, we did not conduct whole exome sequencing on these samples.

Our case series suggests that histologic patterns 1 and 2 may portend more indolent behavior in nTFH cell lymphoma and AITL. A previous study reported [13] that patients with histological pattern 1 may have better outcomes compared to the other 2 patterns. In our case series, the patient with the shortest survival time had histological pattern 3, while patients with histological patterns 1 and 2 were alive at follow-up/died from complications unrelated to nTFH cell lymphoma. Further large-scale studies are required to investigate the prognostic implication of the histological patterns on survival. No patient with bone marrow evaluation had involvement, which may prognosticate survival in this heterogeneous disease. Although nTFH cell lymphomas usually present in the advanced stages [14], 6/9 of the patients in this case series had early-stage disease, which could also explain the favorable outcomes observed.

We propose that not all patients with nTFH cell lymphoma require multiagent chemotherapy in first-line treatment and some can have excellent long-term outcomes. Further research should identify patients with nTFH cell lymphoma of an indolent nature and determine if deferring initial treatment can lead to similar or even better outcomes, as seen in other lymphomas such as mantle-cell lymphoma [15].

## Supplementary information

Supplementary Material

## Data Availability

Data used for analysis can be made available upon reasonable request.
