# Supplementary material for: Indolent nodal T follicular helper cell lymphomas—A case series
Source: Blood Cancer J. 2024 Nov 26;14(1):205. doi: 10.1038/s41408-024-01163-y (PMC11599722; doi:10.1038/s41408-024-01163-y)
Supplement: Supplementary file 1 — Supplementary Material [file 41408_2024_1163_MOESM1_ESM.docx]

# Supplementary Material Legend

Supplementary Table 1: Targeted genes screened for mutations

Supplementary Table 2: Mutations identified in formalin-fixed paraffin-embedded tumor tissue from three subjects

Supplementary Table 3: Tumor Purity from the VAF of the mutations

# Supplementary Table 1: Targeted genes screened for mutations

| Targeted genes screened for mutations | | | | | | | |
| --- | --- | --- | --- | --- | --- | --- | --- |
| ABCA13 | CD74 | DOCK10 | GTF2I | MAML2 | NOTCH3 | PTPRS | TAL2 |
| ABCA4 | CD79A | DOCK3 | HERPUD1 | MAP2 | NPM1 | PTPRT | TANC2 |
| ABCA9 | CD79B | DOCK4 | HIP1 | MAP2K1 | NR4A3 | PVT1 | TBC1D88 |
| ABCB4 | CDC42 | DOCK5 | HIST1H3J | MAP2K4 | NRAS | PXDN | TBX15 |
| ABCC9 | CDC73 | DOCK7 | HIST1H4I | MAPK1 | NRG1 | RABEP1 | TCEA1 |
| ABI1 | CDH1 | DPP6 | HIVEP2 | MAPK3 | NRXN3 | RAC1 | TCF12 |
| ABL1 | CDH11 | DST | HLF | MAPK8 | NSD1 | RAF1 | TCF3 |
| ABL2 | CDK11B | DUSP16 | HMCN1 | MAPK9 | NTRK1 | RANBP17 | TCL1A |
| ACSL3 | CDK4 | DUSP22 | HMGA1 | MAST1 | NTRK3 | RAP1GDS1 | TCL6 |
| ACSL6 | CDK6 | DYRK2 | HMGA2 | MB21D1 | NUMA1 | RARA | TCOF1 |
| ACSM4 | CDKN1B | DYRK3 | HMGN2P46 | MDM2 | NUP214 | RB1 | TDRD15 |
| ADAMTS16 | CDKN2A | ECSIT | HNF1A | MDM4 | NUP98 | RBFOX1 | TECTA |
| ADAMTS20 | CDKN2C | EGFR | HNRNPA2B1 | MDN1 | OLIG2 | RBM15 | TET1 |
| ADGRB1 | CDX2 | EIF4A1 | HOOK3 | MDS2 | OMD | RECQL4 | TET2 |
| ADGRV1 | CEBPA | EIF4A2 | HOXA11 | MECOM | OTOG | REL | TEX15 |
| AFF1 | CEP170 | ELF4 | HOXA13 | MEIOC | P2RY8 | RELN | TFE3 |
| AFF3 | CFAP47 | ELK4 | HOXA9 | MEN1 | PAFAH1B2 | RET | TFEB |
| AFF4 | CHCHD7 | ELL | HOXC11 | MET | PALB2 | RFC2 | TFG |
| AK9 | CHEK2 | ELN | HOXC13 | MGA | PAPPA2 | RGS3 | TFPT |
| AKAP9 | CHIC2 | EML4 | HOXD11 | MGAM | PATZ1 | RGS7 | TFRC |
| AKT1 | CHN1 | ENAM | HOXD13 | MGAM2 | PAX3 | RHOA | THRAP3 |
| AKT2 | CHPF2 | EP300 | HRAS | MITF | PAX5 | RHOH | THSD7A |
| ALDH2 | CHST10 | EPHA6 | HSP90AA1 | MKL1 | PAX7 | RNF213 | TLX1 |
| ALK | CHST6 | EPS15 | HSP90AB1 | MLF1 | PAX8 | ROBO1 | TLX3 |
| ANKRD11 | CHSY3 | ERBB2 | ICOS | MLH1 | PBRM1 | ROBO2 | TMEM173 |
| ANKRD50 | CIC | ERC1 | IDH1 | MLL | PBX1 | ROS1 | TMPRSS2 |
| APC | CIITA | ERCC2 | IDH2 | MLLT1 | PCDH17 | RPL22 | TNFAIP3 |
| APLNR | CILP2 | ERCC3 | IFNAR2 | MLLT10 | PCDH18 | RPN1 | TNFRSF17 |
| ARHGAP26 | CLEC14A | ERCC4 | IFT140 | MLLT11 | PCDH9 | RTN1 | TNS1 |
| ARHGEF12 | CLP1 | ERCC5 | IGH | MLLT3 | PCLO | RUNX1 | TOP1 |
| ARID1A | CLTC | ERG | IGK | MLLT4 | PCM1 | RUNX1T1 | TP53 |
| ARID1B | CLTCL1 | ETV1 | IGL | MLLT6 | PCMTD1 | RYR1 | TPM3 |
| ARID2 | CMPK1 | ETV4 | IGSF1 | MN1 | PCNT | RYR2 | TPM4 |
| ARNT | CMTM4 | ETV5 | IGSF5 | MNX1 | PCSK7 | RYR3 | TPR |
| ASPSCR1 | CMTM6 | ETV6 | IKBKB | MPL | PDCD1LG2 | SBDS | TPRX1 |
| ASXL1 | CMYA5 | EWSR1 | IKZF1 | MROH2B | PDE10A | SCAF8 | TRA |
| ASXL3 | CNBP | EXT1 | IL2 | MSC | PDE4D | SCN1A | TRAF5 |
| ATF1 | CNNM1 | EXT2 | IL21R | MSH2 | PDE4DIP | SCN3A | TRAF6 |
| ATIC | CNTLN | EYS | IL6ST | MSH3 | PDGFB | SDHAF2 | TRANK1 |
| ATM | CNTN3 | FAM123B | IRF4 | MSH6 | PDGFRA | SDHB | TRB |
| ATP10A | CNTRL | FANCA | ITGB4 | MSI2 | PDGFRB | SDHC | TRD |
| ATP10B | COL11A1 | FANCC | ITK | MSN | PDPK1 | SDHD | TRIM24 |
| B2M | COL1A1 | FANCD2 | JAK1 | MTAP | PER1 | SDK1 | TRIM27 |
| BAI3 | COL1A2 | FANCE | JAK2 | MTCL1 | PHOX2B | SDK2 | TRIM33 |
| BANK1 | COL4A1 | FANCF | JAK3 | MTCP1 | PICALM | SEMA3C | TRIO |
| BCL10 | COX6C | FANCG | JAZF1 | MUC1 | PIK3CA | SEPT5 | TRIP11 |
| BCL11A | CREB1 | FAS | JUN | MUC16 | PIK3CB | SEPT6 | TRPC6 |
| BCL11B | CREB3L2 | FAT4 | KCND2 | MUTYH | PIK3CG | SEPT9 | TSC1 |
| BCL2 | CREBBP | FBN2 | KCNH2 | MYB | PIK3R1 | SET | TSC2 |
| BCL3 | CREBL2 | FBXW7 | KCNMA1 | MYC | PIK3R5 | SETD2 | TSHR |
| BCL5 | CRLF2 | FCGR2B | KDM5A | MYCBP2 | PIM1 | SFPQ | TTC6 |
| BCL6 | CRTC1 | FCRL4 | KDM5B | MYCL1 | PIP4K2C | SH3GL1 | TTL |
| BCL7A | CRTC3 | FEV | KDM5C | MYCN | PKHD1 | SHANK1 | TYK2 |
| BCL9 | CSMD1 | FEZF1 | KDM6A | MYD88 | PKHD1L1 | SI | TYR |
| BCOR | CSMD3 | FGFR1 | KDR | MYH1 | PLAG1 | SIPA1L3 | UGGT2 |
| BCR | CTLA4 | FGFR1OP | KDSR | MYH11 | PLCE1 | SLC17A6 | UNC13C |
| BIRC3 | CTNNB1 | FGFR2 | KIAA1549 | MYH15 | PLCG1 | SLC45A3 | UNC5D |
| BLM | CTNND1 | FGFR3 | KIF1B | MYH2 | PLCG2 | SLC5A11 | USP6 |
| BMPR1A | CXCR7 | FH | KIT | MYH4 | PLCH1 | SLIT2 | VAV1 |
| BRAF | CYLC1 | FIP1L1 | KLF6 | MYH9 | PLCZ1 | SLITRK1 | VAV2 |
| BRCA1 | CYLD | FLI1 | KLK2 | MYO18B | PLXNA1 | SMAD4 | VAV3 |
| BRCA2 | DCC | FLT3 | KMT2D | MYO9A | PLXNA2 | SMARCA4 | VCAN |
| BRD3 | DDB2 | FNBP1 | KRAS | MYT1L | PLXNA4 | SMARCB1 | VHL |
| BRD4 | DDIT3 | FOXL2 | KTN1 | N4BP1 | PLXNB2 | SMCHD1 | VLDLR |
| BRIP1 | DDX10 | FOXO1 | LAMA1 | NACA | PML | SMO | VPS13A |
| BTG1 | DDX25 | FOXO3 | LAMA5 | NALCN | PMS1 | SOCS1 | WDFY3 |
| BUB1B | DDX3X | FOXO4 | LASP1 | NAV2 | PMS2 | SORBS2 | WDR66 |
| C2orf16 | DDX5 | FOXP1 | LAT | NBEA | PNLIP | SPECC1 | WHSC1 |
| CACNA1B | DDX55 | FREM2 | LATS2 | NBN | POU2AF1 | SRGAP3 | WHSC1L1 |
| CACNA1E | DDX58 | FSIP2 | LCK | NCAM2 | POU5F1 | SRSF3 | WRN |
| CANT1 | DDX6 | FSTL3 | LCP1 | NCKAP5 | PPARG | SS18 | WT1 |
| CARD11 | DDX60L | FUS | LHFP | NCKIPSD | PPP1R3A | SS18L1 | XIRP2 |
| CARS | DEK | FYN | LHX3 | NCOA1 | PRCC | SSC5D | XPA |
| CASC5 | DENND5B | GABRA5 | LIFR | NCOA2 | PRDM1 | SSX1 | XPC |
| CBFA2T3 | DHX30 | GABRG2 | LINGO2 | NCOA4 | PRDM16 | SSX2 | ZAP70 |
| CBFB | DHX58 | GABRR1 | LMCD1 | NEB | PRF1 | SSX4 | ZBTB16 |
| CBLB | DHX9 | GAS7 | LMO1 | NEFH | PRKCQ | STAT3 | ZFHX4 |
| CBLC | DIAPH2 | GATA1 | LMO2 | NEK2 | PRKD1 | STAT4 | ZMYM2 |
| CC2D2A | DICER1 | GATA2 | LOC100128317 | NEK6 | PRMT5 | STAT5B | ZNF331 |
| CCDC168 | DLGAP2 | GATA3 | LPP | NF1 | PRRX1 | STIL | ZNF384 |
| CCDC185 | DLGAP3 | GFRAL | LRP1 | NF2 | PRUNE2 | STK11 | ZNF462 |
| CCDC6 | DMBT1 | GMPS | LRP1B | NFIB | PSIP1 | STL | ZNF521 |
| CCNB1IP1 | DMD | GNAI2 | LRP6 | NFKB2 | PTCH1 | SUFU | ZNRF3 |
| CCND1 | DMXL2 | GNAQ | LRRK2 | NIN | PTEN | SUN1 |  |
| CCND2 | DNAH14 | GNAS | LYL1 | NLRP11 | PTPN11 | SUZ12 |  |
| CCND3 | DNAH5 | GOLGA5 | M6PR | NLRP4 | PTPRC | SVIL |  |
| CD274 | DNAH7 | GOPC | MAF | NOL4 | PTPRD | SYK |  |
| CD279 | DNAH8 | GPHN | MAFB | NONO | PTPRG | SYNE1 |  |
| CD28 | DNAH9 | GRIN2B | MALAT1 | NOTCH1 | PTPRM | TAF15 |  |
| CD36 | DNMT3A | GSK3β | MALT1 | NOTCH2 | PTPRQ | TAL1 |  |

# Supplementary Table 2: Mutations identified in formalin-fixed paraffin-embedded tumor tissue from three subjects

| Case number | Sequencing_Sample_ID | Gene symbols | Variant_depth | Total_depth | Variant allele frequency | Splicing details | Type of exonic change | Nucleotide change in codon and amino acid change in protein peptide chain |
| --- | --- | --- | --- | --- | --- | --- | --- | --- |
| Case 1 | PLB8AI174 | DOCK7 | 3 | 17 | 0.17647059 |  | nonsynonymous SNV | DOCK7:NM_001271999:exon11:c.G1282A:p.E428K,DOCK7:NM_001272000:exon11:c.G1282A:p.E428K,DOCK7:NM_001272001:exon11:c.G1282A:p.E428K,DOCK7:NM_001272002:exon11:c.G1282A:p.E428K,DOCK7:NM_001330614:exon11:c.G1282A:p.E428K,DOCK7:NM_033407:exon11:c.G1282A:p.E428K |
| Case 1 | PLB8AI174 | MYT1L | 4 | 28 | 0.14285714 |  | nonsynonymous SNV | MYT1L:NM_001303052:exon9:c.G334A:p.E112K,MYT1L:NM_001329845:exon9:c.G334A:p.E112K,MYT1L:NM_001329846:exon9:c.G334A:p.E112K,MYT1L:NM_001329847:exon9:c.G334A:p.E112K,MYT1L:NM_001329848:exon9:c.G334A:p.E112K,MYT1L:NM_001329849:exon9:c.G334A:p.E112K,MYT1L:NM_001329851:exon9:c.G334A:p.E112K,MYT1L:NM_001329852:exon9:c.G334A:p.E112K,MYT1L:NM_015025:exon9:c.G334A:p.E112K,MYT1L:NM_001329844:exon10:c.G334A:p.E112K |
| Case 1 | PLB8AI174 | PIK3CA | 3 | 22 | 0.13636364 | NM_006218:exon16:c.2416+1G>A | splicing |  |
| Case 1 | PLB8AI174 | LPP | 71 | 164 | 0.43292683 |  | nonsynonymous SNV | LPP:NM_001167672:exon2:c.G65A:p.R22Q,LPP:NM_001167671:exon3:c.G65A:p.R22Q,LPP:NM_005578:exon3:c.G65A:p.R22Q |
| Case 1 | PLB8AI174 | VPS13A | 11 | 22 | 0.5 |  | nonsynonymous SNV | VPS13A:NM_001018037:exon53:c.A7495C:p.N2499H,VPS13A:NM_001018038:exon54:c.A7612C:p.N2538H,VPS13A:NM_015186:exon54:c.A7612C:p.N2538H,VPS13A:NM_033305:exon54:c.A7612C:p.N2538H |
| Case 1 | PLB8AI174 | TET1 | 4 | 15 | 0.26666667 |  | nonsynonymous SNV | TET1:NM_030625:exon11:c.G5348A:p.R1783Q |
| Case 1 | PLB8AI174 | PICALM | 12 | 23 | 0.52173913 |  | nonsynonymous SNV | PICALM:NM_001206946:exon13:c.G1307A:p.S436N,PICALM:NM_007166:exon13:c.G1328A:p.S443N |
| Case 1 | PLB8AI174 | DCC | 23 | 55 | 0.41818182 |  | nonsynonymous SNV | DCC:NM_005215:exon27:c.C4108A:p.P1370T |
| Case 1 | PLB8AI174 | SEPT5 | 3 | 18 | 0.16666667 |  | nonsynonymous SNV | SEPT5:NM_001009939:exon3:c.G257A:p.S86N,SEPT5:NM_002688:exon4:c.G230A:p.S77N |
| Case 1 | PLB8AI174 | DMD | 4 | 30 | 0.13333333 |  | nonsynonymous SNV | DMD:NM_004014:exon2:c.G182A:p.R61Q,DMD:NM_004013:exon13:c.G989A:p.R330Q,DMD:NM_004020:exon13:c.G989A:p.R330Q,DMD:NM_004021:exon13:c.G989A:p.R330Q,DMD:NM_004022:exon13:c.G989A:p.R330Q,DMD:NM_004023:exon13:c.G989A:p.R330Q,DMD:NM_004011:exon28:c.G4346A:p.R1449Q,DMD:NM_004012:exon28:c.G4337A:p.R1446Q,DMD:NM_000109:exon56:c.G8345A:p.R2782Q,DMD:NM_004006:exon56:c.G8369A:p.R2790Q,DMD:NM_004009:exon56:c.G8357A:p.R2786Q,DMD:NM_004010:exon56:c.G8000A:p.R2667Q |
| Case 1 | PLB8AI174 | PKHD1L1 | 18 | 86 | 0.20930233 |  | nonsynonymous SNV | PKHD1L1:NM_177531:exon36:c.G4442A:p.S1481N |
| Case 1 | PLB8AI174 | PCLO | 76 | 151 | 0.50331126 |  | nonframeshift insertion | PCLO:NM_014510:exon2:c.1125_1126insCCTCCAGCTCAGCAC:p.Q375_T376insPPAQH,PCLO:NM_033026:exon2:c.1125_1126insCCTCCAGCTCAGCAC:p.Q375_T376insPPAQH |
| Case 1 | PLB8AI174 | PCLO | 79 | 145 | 0.54482759 |  | nonframeshift insertion | PCLO:NM_014510:exon2:c.1123_1124insCTCTTGGTCCTGCTA:p.Q375delinsPLGPAK,PCLO:NM_033026:exon2:c.1123_1124insCTCTTGGTCCTGCTA:p.Q375delinsPLGPAK |
| Case 2 | PLB8AI173 | PBRM1 | 4 | 28 | 0.14285714 |  | nonsynonymous SNV | PBRM1:NM_001350075:exon24:c.G3875A:p.R1292K,PBRM1:NM_018313:exon24:c.G3800A:p.R1267K,PBRM1:NM_181042:exon25:c.G3875A:p.R1292K,PBRM1:NM_001350079:exon26:c.G3863A:p.R1288K,PBRM1:NM_001350074:exon27:c.G3983A:p.R1328K,PBRM1:NM_001350076:exon27:c.G3980A:p.R1327K,PBRM1:NM_001350078:exon27:c.G3983A:p.R1328K,PBRM1:NM_001350077:exon28:c.G3974A:p.R1325K |
| Case 2 | PLB8AI173 | RANBP17 | 4 | 32 | 0.125 |  | nonsynonymous SNV | RANBP17:NM_022897:exon14:c.C1669T:p.R557C |
| Case 2 | PLB8AI173 | DNAH8 | 3 | 16 | 0.1875 |  | nonsynonymous SNV | DNAH8:NM_001206927:exon82:c.C12265T:p.R4089C |
| Case 2 | PLB8AI173 | AK9 | 80 | 204 | 0.39215686 |  | nonsynonymous SNV | AK9:NM_001145128:exon41:c.G5652C:p.K1884N |
| Case 2 | PLB8AI173 | BIRC3 | 4 | 27 | 0.14814815 |  | nonsynonymous SNV | BIRC3:NM_001165:exon7:c.C1376T:p.T459I,BIRC3:NM_182962:exon8:c.C1376T:p.T459I |
| Case 2 | PLB8AI173 | DICER1 | 18 | 50 | 0.36 |  | nonsynonymous SNV | DICER1:NM_001195573:exon22:c.C4991T:p.S1664L,DICER1:NM_001271282:exon23:c.C4991T:p.S1664L,DICER1:NM_001291628:exon23:c.C4991T:p.S1664L,DICER1:NM_177438:exon23:c.C4991T:p.S1664L,DICER1:NM_030621:exon25:c.C4991T:p.S1664L |
| Case 2 | PLB8AI173 | FANCA | 71 | 154 | 0.46103896 |  | nonsynonymous SNV | FANCA:NM_000135:exon28:c.A2660G:p.D887G,FANCA:NM_001286167:exon28:c.A2660G:p.D887G |
| Case 3 | PLB8AI177 | DNAH14 | 42 | 95 | 0.44210526 |  | nonsynonymous SNV | DNAH14:NM_001373:exon27:c.T4400C:p.I1467T |
| Case 3 | PLB8AI177 | AFF1 | 20 | 44 | 0.45454545 |  | nonsynonymous SNV | AFF1:NM_001313960:exon10:c.C1463G:p.T488R,AFF1:NM_001313959:exon12:c.C2549G:p.T850R,AFF1:NM_005935:exon12:c.C2549G:p.T850R,AFF1:NM_001166693:exon13:c.C2570G:p.T857R |
| Case 3 | PLB8AI177 | BRCA2 | 3 | 17 | 0.17647059 |  | nonsynonymous SNV | BRCA2:NM_000059:exon11:c.G2491A:p.V831I |

Raw sequencing reads were aligned to the hs37d5 reference genome using BWA MEM(1). Alignments were sort and PCR duplicated were removed by Sambamba (v0.6.5)(2). Strelka2 (v2.9.4)(3) was used to call the candidate variants from the aligned sequencing data and annotated was done by wAnnovar(4) on 6 January 2022. Final list of variants also have to pass through various blacklist filters(5), minor-allele frequency of < 1%, variant read-depth >= 3x, variant allele frequency > 5% and cannot be identified with a dbSNP ID without an COSMIC ID to avoid germline bleed-through. Highly repetitive positional variants without COSMIC IDs were also filtered.

# Supplementary Table 3: Tumor Purity from the VAF of the mutations

| **SampleID** | **Number of considered SNVs** | **Average of VAF** | **Purity Estimate** |
| --- | --- | --- | --- |
| PLB8AI172 | 44 | 0.41917146 | 0.83834293 |
| PLB8AI173 | 49 | 0.34362384 | 0.68724768 |
| PLB8AI174 | 55 | 0.32372006 | 0.64744012 |
| PLB8AI175 | 43 | 0.38173097 | 0.76346195 |
| PLB8AI177 | 42 | 0.47350822 | 0.94701643 |

# References

1. Li H. Aligning sequence reads, clone sequences and assembly contigs with BWA-MEM. arXiv:1303. 2013.

2. Tarasov A, Vilella AJ, Cuppen E, Nijman IJ, Prins P. Sambamba: fast processing of NGS alignment formats. Bioinformatics. 2015;31(12):2032-4.

3. Kim S, Scheffler K, Halpern AL, Bekritsky MA, Noh E, Kallberg M, et al. Strelka2: fast and accurate calling of germline and somatic variants. Nature methods. 2018;15(8):591-4.

4. Chang X, Wang K. wANNOVAR: annotating genetic variants for personal genomes via the web. J Med Genet. 2012;49(7):433-6.

5. Lim JQ, Huang D, Chan JY, Laurensia Y, Wong EKY, Cheah DMZ, et al. A genomic-augmented multivariate prognostic model for the survival of natural-killer/T-cell lymphoma patients from an international cohort. Am J Hematol. 2022;97(9):1159-69.
